# Immunoproteomic Analysis of Human Serological Antibody Responses to Vaccination with Whole-Cell Pertussis Vaccine (WCV)

**DOI:** 10.1371/journal.pone.0013915

**Published:** 2010-11-09

**Authors:** Yong-Zhang Zhu, Cheng-Song Cai, Wei Zhang, Hong-Xiong Guo, Jin-Ping Zhang, Ya-Yong Ji, Guang-Yuan Ma, Jia-Lin Wu, Qing-Tian Li, Cheng-Ping Lu, Xiao-Kui Guo

**Affiliations:** 1 Department of Medical Microbiology and Parasitology, Institutes of Medical Sciences, Shanghai Jiao Tong University School of Medicine, Shanghai, People's Republic of China; 2 Molecular Biology Laboratory, WuXi Center For Disease Prevention and Control, WuXi, People's Republic of China; 3 Key Laboratory of Animal Disease Diagnostic and Immunology, Ministry of Agriculture, Nanjing Agricultural University, Nanjing, People's Republic of China; 4 Department of STD and AIDS Prevention and Control, Jiangsu Center For Disease Prevention and Control, Nanjing, People's Republic of China; 5 Department of Laboratory Medicine, Ruijin Hospital, Shanghai Jiao Tong University School of Medicine, Shanghai, People's Republic of China; Charité-Universitätsmedizin Berlin, Germany

## Abstract

**Background:**

Pertussis (whooping cough) caused by *Bordetella pertussis* (*B.p*), continues to be a serious public health threat. Vaccination is the most economical and effective strategy for preventing and controlling pertussis. However, few systematic investigations of actual human immune responses to pertussis vaccines have been performed. Therefore, we utilized a combination of two-dimensional electrophoresis (2-DE), immunoblotting, and mass spectrometry to reveal the entire antigenic proteome of whole-cell pertussis vaccine (WCV) targeted by the human immune system as a first step toward evaluating the repertoire of human humoral immune responses against WCV.

**Methodology/Principal Findings:**

Immunoproteomic profiling of total membrane enriched proteins and extracellular proteins of Chinese WCV strain 58003 identified a total of 30 immunoreactive proteins. Seven are known pertussis antigens including Pertactin, Serum resistance protein, chaperonin GroEL and two OMP porins. Sixteen have been documented to be immunogenic in other pathogens but not in *B.p*, and the immunogenicity of the last seven proteins was found for the first time. Furthermore, by comparison of the human and murine immunoproteomes of *B.p*, with the exception of four human immunoreactive proteins that were also reactive with mouse immune sera, a unique group of antigens including more than 20 novel immunoreactive proteins that uniquely reacted with human immune serum was confirmed.

**Conclusions/Significance:**

This study is the first time that the repertoire of human serum antibody responses against WCV was comprehensively investigated, and a small number of previously unidentified antigens of WCV were also found by means of the classic immunoproteomic strategy. Further research on these newly identified predominant antigens of *B.p* exclusively against humans will not only remarkably accelerate the development of diagnostic biomarkers and subunit vaccines but also provide detailed insight into human immunity mechanisms against WCV. In particular, this work highlights the heterogeneity of the *B.p* immunoreactivity patterns of the mouse model and the human host.

## Introduction

*Bordetella pertussis* (*B.p*) is a strictly obligate human pathogen and the causative agent of a seriously contagious childhood respiratory disease, whooping cough or pertussis, which causes 300,000 children death mainly in developing countries and afflicts up to 40 million children worldwide per year [1]. Vaccination is the most economical and effective strategy for preventing and controlling pertussis. The introduction of the first generation of pertussis vaccines in the 1950s dramatically reduced the incidence of the disease [2]. Now, WCV and acellular pertussis vaccines (ACV) are two main types of pertussis vaccines that are used globally [3]. Despite the high vaccination coverage all over the world, pertussis is still a serious contagious childhood acute respiratory disease, especially in infants less than 6 months old [4]. Frequent pertussis outbreaks have been reported recently [5]. Recent investigations have revealed that even older children, adolescents and adults immunized with the vaccine or infected previously could also be infected by the disease again and in turn act as important sources of transmission to young infants who are either non-vaccinated or too young to be vaccinated [6], [7].

Unlike other gram-negative pathogens, diphtheria, tetanus or Hepatitis B virus, the pathogenesis of *B.p* is much more complex because a range of different virulence determinants have been implicated [8]. *B.p* exists in three distinct phenotypes, virulent Bvg+ (Bordetella virulence gene) phase, avirulent Bvg- phase and Bvg-intermediate phase (Bvgi) controlled by the BvgAS two-component signal transduction system. Each of the three phases is classically characterized by the maximal expression of a subset of Bvg phase-specific genes. These key virulence determinants of *B.p* are divided into two main groups: adhesins, such as Filamentous hemagglutinin (FHA), Peracitin (Prn), Fimbriae 2 and 3, Serum resistance protein (BrkA), Tracheal colonization factor (TcfA); and toxins, such as Pertussis toxin (PT), Tracheal cytotoxin (TCT), Dermonecrotic toxin (DNT) and Adenylate cyclase (CyaA). Almost all of the known virulence determinants are virulent Bvg+ phase-specific genes. Numerous investigations of these virulence determinants have vastly contributed to our understanding of immunity mechanisms against *B. p* infection and immunization with pertussis vaccines [8]; however, the basis of the protective immunity of these identified virulence factors is not fully understood, and some unknown antigens remain to be further investigated. Therefore, the exact immunity mechanism of *B. p* in human hosts is still far from clear.

It should be noted that *B. p* is a strictly obligate human pathogen with no known animal and environmental reservoir; experimental infection of animal models only occurs when these animals are immunized with large inoculating doses of *B. p*. Compared to pertussis patients, most animal models of *B.p* produce different clinical symptoms, including lack of cough, symptomatic upper respiratory infection and whoop, and the consistent production of pertussis pneumonia [9], [10]. Hence, there may be many differences between human hosts and animal models. Furthermore, paroxysmal cough, the most characteristic symptom of pertussis infected infants, is not observed in most animal models [10]. Additionally, based on the strikingly converse serum antibody responses between mice and children immunized with WCV against many main *B.p* protective antigens such as PT and FHA, an earlier investigation has shown that the murine model as a main pertussis animal model should not be globally applied to evaluate the protective efficacy of pertussis vaccines comprising these antigenic components [11]. Thus, these evidences suggest that these animal models are limited in their degree of sensitivity to accurately reflect events occurring during pertussis infection or vaccination in human hosts. Therefore, there is a growing effort to elucidate human immune responses against *B. p* infection and immunization with pertussis vaccines.

A large numbers of investigations have utilized immunoproteomic technology, combining 2-DE with immunoblot analysis of antigenic proteins, which are globally applied to reveal immune responses of the host against pathogen antigens as well as to identify suitable prognostic and diagnostic biomarkers and pathogenic targets for developing new drugs and vaccines [12], [13], [14], [15], [16], [17], [18], [19], [20]. According to the theory of reverse vaccinology, total membrane enriched proteins (TMPs) and extracellular proteins (ECPs) of bacterial pathogens primarily mediate many critical biological cellular processes, including host-pathogen interactions, virulence and pathogenesis, survival of the pathogen in the intracellular environment and the evasion of the host immune system, and are frequently main targets of the host immune system; hence, these proteins are certainly useful candidate antigens for vaccine and diagnostic development [21], [22], [23]. Thus, it is remarkably valuable to screen suitable vaccine candidates from these two sub-proteomes of *B. p*. Recently, systematic identification of antigenic proteins from the whole proteome of *B. p* in infected or immunized mouse serum has been reported [20]. However, a full understanding of the repertoire of human immune responses against pertussis has been limited because of a lack of detailed knowledge of the entire antigenic composition of WCV recognized by human hosts. In the present study, we used a classic immunoproteomic strategy to investigate the complete set of antigenic proteins from TMP and ECP preparations of Chinese WCV strain 58003 and have provided a complete immunoproteomic reference map for future studies of *B. p*. This report is the first to precisely characterize the repertoire of human serum antibody responses against WCV, and it will open the door toward the downselection of ideal protective antigens for incorporation into pertussis subunit vaccines. Finally, the data obtained from our study will significantly accelerate the development of new diagnostic markers and a new generation of *B. p* subunit vaccines.

## Materials and Methods

### 2.1 Ethics Statement

The study protocol was approved by the Ethics Committee of Shanghai Jiao Tong University and conforms to the principles outlined in the Declaration of Helsinki. The ten hyper-immune serum samples from children immunized with WCV that were kindly donated by Professor Zhang Shu-Min (Division of Serum, National Institute for Control of Pharmaceutical and Biological Product) were pooled together. Negative control sera were randomly obtained from two unvaccinated infants in one local children's hospital with waiver of consent, which the Ethics Committee of Shanghai Jiao Tong University approved. However, because of the requirement of these participants' parents, nearly all of serum samples were anonymous and no personal information was collected. Thus, written, informed consent was not obtained from these participants.

### 2.2 Preparation of TMPs and ECPs of *B.p* Chinese WCV strain 58003 for 2-DE

High-density whole cell culture and culture supernatant of Chinese WCV strain 58003 (6×10^9^ cell/ml) were kindly provided by Xiang Mei-Juan (Department of Pertussis Whole-Cell Vaccines, Wuhan Institute of Biologic Products). The Chinese WCV strain 58003 was grown in Stainer-Scholte liquid medium (protein-free) at 37°C. Culture supernatant was collect by centrifugation for 15 min at 4,000×g at 4°C and filtered through a 0.22 µm membrane to remove residual bacteria. Then, the filtrate was treated with chilled 15% TCA in an ice-bath for 30 min to precipitate proteins. After centrifugation at 10,000×g for 10 min at 4°C, the precipitated proteins were washed three times with chilled acetone to remove TCA and were naturally air-dried. Finally, the ECPs were stored at −80°C for subsequent analysis.

TMPs of Chinese WCV strain 58003 were extracted as previously described by Zhang et al [16]. In brief, the bacterial cell pellet was resuspended in solution A containing 80 mm Tris–HCl, pH 7.4 and 1.2 M NaCl and sonicated in an ice-bath. The solution was centrifuged at 10,000×g for 20 min to remove unbroken cells and debris. Next, one-third volume of solution B containing 40 mM Tris–HCl, pH 7.4, 600 mM NaCl and 4% Triton X-114 (Amresco, USA) was added, followed by incubation on ice for 1 h with frequent vortexing. After incubation at 30°C for 3 min, the sample produced an upper aqueous phase and a lower detergent phase by centrifugation at 1300×g for 10 min at room temperature. TMPs in the detergent phase were precipitated with 10 volumes of chilled acetone overnight at −20°C. By centrifugation at 10000×g for 10 min, TMPs were naturally air-dried and were stored at −80°C for subsequent analysis.

Prior to 2-DE, TMPs and ECPs were both further purified by using a 2-DE clean-up kit (GE Healthcare).

### 2.3 2DE-PAGE

2DE-PAGE was performed as previously described by zhang et al [16], [19]. 7 cm Immobiline DryStrip (IPG, pH 4–7; Bio-Rad) dissolving 150 µg ECPs in a total volume of 150 µL rehydration buffer (8 M urea, 2% CHAPS, 50 mM DTT, 0.2% Bio-Lyte 4–7 Ampholyte, 0.002% Bromophenol Blue; Bio-Rad) was rehydrated for 13 h at 20°C and IEF was performed in a PROTEAN IEF cell (Bio-Rad) under the running conditions as following: 250 V for 0.5 h, 500 V for 0.5 h, 4000 V for 3 h, and 4000 V for 20000 Vh. Similarly, 13 cm Immobiline DryStrip (IPG, pH 4–7; GE Healthcare) dissolving 350 µg TMPs in a total volume of 250 µL rehydration buffer (7 M urea, 2 M thiourea, 2% CHAPS, 50 mM DTT, 0.2% Bio-Lyte 4–7 Ampholyte, 0.002% bromophenol blue; Bio-Rad) was rehydrated for 14 h at 20°C and IEF was performed in a Multiphor II IEF system (GE Healthcare) under the running conditions as following: 500 V for 4 h, 1000 V for 1 h, 2000 V for 1 h, 4000 V for 1 h, and 8000 V for 36000 Vh. After electrophoresis, each IPG strip was washed for 15 min in equilibration buffer A (375 mM Tris-HCl, 6 M urea, 2% SDS, 2% w/v DTT; Bio-Rad) and in equilibration buffer B (375 mM Tris-HCl, 6 M urea, 2% SDS, 2.5% w/v iodoacetamide; Bio-Rad). The IPG strips were then placed onto a 12.5% SDS-PAGE gel and the second dimensional separation was performed in two steps at 10°C: 80 V/gel for 30 min and 120 V/gel until the tracking dye reached the bottom of the gels. One gel used as a reference proteome map was Coomassie blue stained with CBB-G250, and the other duplicated gel was transferred onto PVDF membrane for subsequent immunoblot analysis.

### 2.4 Immunoblot analysis of 2-DE

The 2DE-PAGE separated proteins were electroblotted onto PVDF membrane (GE Healthcare) using a semidry transfer system (Hoefer TE 77, GE Healthcare) for 1 h with a current of 1 mA/cm^2^. The PVDF membranes were blocked with 5% w/v defatted milk in TBS-T (50 mM Tris-Cl, pH 7.4, 200 mM NaCl, 0.05% w/v Tween-20) for 2 h at room temperature. Then, the membranes were incubated with a 1∶1000 dilution of pooled serum at 4°C overnight and subsequently washed four times with TBS-T for 5 min. Next, the membranes were further incubated with a 1∶2000 dilution of peroxidase-labeled rabbit anti-human IgG (Jackson, USA) for 1 h at room temperature. Finally, the membranes were washed five times with TBS-T for 5 min and developed with 3,3′-diaminobenzidine (DAB, Sigma) substrate until optimum color development was observed. Each immunoblotting experiment was performed in duplicate.

### 2.5 MALDI-TOF-MS analysis and database search

Spots were excised from the 2D coomassie blue-stained gels and sent to Shanghai GeneCore BioTechnologies Co., Ltd for tryptic in-gel digestion and MALDI-TOF-MS with a Voyager DE Pro MALDI-TOF mass spectrometer (ABI). Peptide mass fingerprint (PMF) data was searched against the NCBI database in MASCOT server (http://www.matrixscience.com) for sequence match. The MASCOT search parameters were as follows: Bacteria (Eubacteria); one missed cleavage by trypsin digestion; fixed posttranslational modification (oxidized methionine); peptide charge (positive); peptide mass tolerance (±100 ppm) and fragment mass tolerance (±0.5 Da). The MASCOT probability score for the match, molecular weight (MW), isoelectric point (pI), number of peptide matches and percentage of the total amino acid sequence covered by the peptides were comprehensively analyzed for confident spot identification. Successful protein identification must meet the following criteria: these peptides defined as significant hit by MASCOT probability analysis and the MASCOT probability score of these peptides for the match higher than 60; at least 6 matching peptides and amino acid sequence coverage more than 20%.

### 2.6 Bioinformatic analysis

In addition to spot identification by PMF, a detailed analysis of these identified immunoreactive proteins was performed using a variety of bioinformatics tools. We adapted a previously developed approach with some modifications to predict subcellular localization of gram-negative bacterial proteins [24]. Protein families were identified using Pfam (http://www.sanger.ac.uk/Software/Pfam/). The theoretical molecular weight and isoelectric point were calculated using the compute pI/MW tool (http://www.expasy.org/tools/pi_tool.html/). The Codon Adaptation Index (CAI) of each protein was evaluated with the JCAT tool (http://www.jcat.de).

### 2.7 Previous proteomic data and transcriptional profiling of other *B.p* vaccine strains

In addition to human immunorpteome of *B.p* Chinese WCV strain 58003 in the present study, Murine immunoproteomes of *B.p* vaccine strains Saadet and Tohama recently performed by Emrah altindis et al were directly obtained from their published study [20]; The outer membrane vesicles (OMV) proteome and Surfaceome of *B.p* vaccine strain Tohama CIP 8132 was derived from Daniela Hozbor et al [25], [26]; Global expression profiling of the *B.p* strain Tohama was obtained from the AarrayExpress database in the EBI (The experimental protocol and data analysis of these microarray are compliant with current MIAME guideline. The detailed MIAME information of these microarray is shown in http://www.ebi.ac.uk/microarray-as/aer/details?class=MAGE.Experiment_protocols&criteria=Experiment%3D531022681&contextClass=MAGE.Protocol&templateName=Protocol.vm), and all raw and processed microarray data are available in the AarrayExpress database. Accession Number: E-TABM-31) [27].

## Results

### 3.1 2-DE proteome profiling of TMPs and ECPs of *B.p* Chinese WCV strain 58003

According to preliminary experiment results, the vast majority of immunoreactive proteins were restricted between pH 4.0 and 7.0 (data not shown). Therefore, our experimental strategy utilized 2-DE and immunoblotting analysis of TMPs and ECPs of WCV strain 58003 with proteins resolved between pH 4.0 and 7.0.

**Figure 1:**
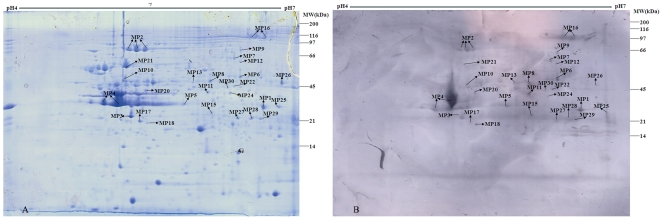
2-D proteome reference map and representative immunoblot of TMPs of *B.pertussis* Chinese WCV strain 58003. TMPs were separated by IEF at pH 4–7 in the first dimension and then by 12.5% SDS-PAGE in the second dimension. Gels were either Coomassie blue-stained (Fig. 1 A) or immunoblotted with a 1∶1000 dilution of pooled immune sera from vaccinated children (Fig. 1B). The protein spots of interest were excised individually for identification by PMF. The spot numbers refer to the identified immunoreactive proteins listed in Table S1.

**Figure 2:**
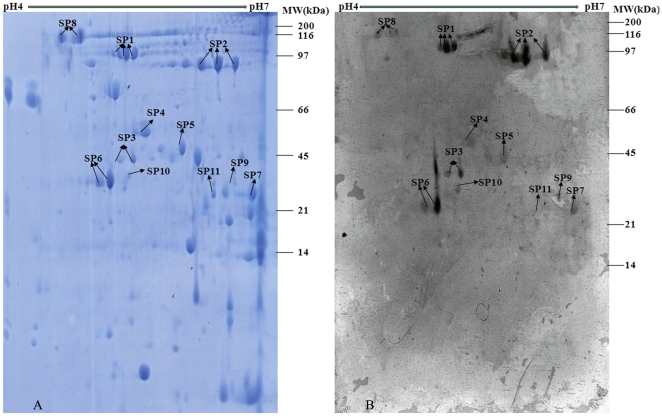
2-D proteome reference map and representative immunoblot of ECPs of *B.pertussis* Chinese WCV strain 58003. ECPs were separated by IEF at pH 4–7 in the first dimension and then by 12.5% SDS-PAGE in the second dimension. Gels were either Coomassie blue-stained (Fig. 2A) or immunoblotted with a 1∶1000 dilution of pooled immune sera from vaccinated children (Fig. 2B). The protein spots of interest were excised individually for identification by PMF. The spot numbers refer to the identified immunoreactive proteins listed in Table S1.

The 2-DE profiles of TMPs and ECPs of *B.p* WCV strain 58003 revealed more than 350 and 60 protein spots, respectively, on the gels with pIs ranging from 4.0 to 7.0 and molecular masses ranging from 10 to 160 kDa (See Fig. 1A–2A). After performing immunoblot analysis, the corresponding immunoreactive protein spots were excised from Coomassie blue-stained gels for protein identification by PMF.

### 3.2 Identification of human immunoreactive proteins from TMPs and ECPs of *B.p* Chinese WCV strain 58003

The application of MALDI-MS approach resulted in successful identification of 41 immunoreactive protein spots corresponding to 30 distinct proteins which included 23 TMPs and 11 ECPs with molecular masses ranging from 29 to 137 kDa. These immunoreactive proteins of *B.p* WCV strain 58003 are listed in Table S1 and their positions are shown on the reference 2D proteome and representative immunoproteome maps (See Fig. 1–2). A few weak reactions were also observed with negative sera, but none of these immunoreactive proteins reacted with the negative sera (See Figure S1). The average experimental molecular mass of all the identified proteins was 49.6 kDa, and their average pI was 5.84. The smallest protein identified was a putative ABC transporter ATP-binding protein of 29.6 kDa, while the largest protein was BipA at 137.1 kDa. The pI of the identified proteins ranged from pI 4.07 for OmpP to pI 7.08 for PBP. In particular, the four proteins Prn, BrkA, OmpP and Sbp were identified as immunogenic proteins in both the TMP and the ECP preparations. As frequently described for many pathogens, multiple different spots in the same gel with distinct charges or molecular masses were often identified as the same protein encoded by a single gene. The most frequent examples in this study were Prn (3 different spots), BrkA (3 spots) and GroEL (2 spots).

## Discussion

Here, by combining the present study with previous murine immunoproteomic studies of *B.p*, we discovered approximately 30 highly specific immunoreactive proteins directed against human hosts. These human immunoreactive proteins of WCV identified in this work can be divided into three main groups (See Table S2). The first group is composed of the seven known immunoreactive antigens including Prn, BrkA, GroEL, BipA, PtlF and two porins, OmpP and OmpQ, which have previously been well-characterized in *B. p*. With the exception of the Bvgi- phase-specific gene BipA, all other known pertussis antigens are virulent Bvg+ phase-specific genes associated with the pathogenesis and virulence of *B. p*. The immunoreactive proteins of the second group can be divided into two subgroups, including those commonly conserved antigenic proteins that have already been shown to be highly immunogenic in more than four distinct pathogenic bacteria such as Ldh, GdhA, SdhA, GAPDH, EF-Tu, EF-Ts and putative ABC transporter ATP-binding protein, and another uniquely antigenic proteins in one (or occasionally several) pathogenic bacteria, such as NuoD, MetC, SucC, Icd, MinD, PBP, LivJ, putative ABC transport solute binding protein and putative alcohol dehydrogenase. Finally, the third group is composed of several previously unidentified immunoreactive proteins, such as Sbp and three hypothetical proteins (BP0250, BP2818 and BP3575). Furthermore, a total of 18 human immunoreactive proteins that include the seven known pertussis antigens and newly identified antigens (putative ABC transport ATP binding protein, putative ABC transport solute binding protein, hypothetical proteins BP2818, PBP, EF-Tu, EF-Ts, Ldh, SucC, LivJ and SdhA) have been previously found in the Surfaceome or OMV proteome of *B. p*, indicating that these surface antigens could serve as predominant targets recognized by the host immune system and, hence, induce strong host immune responses.

However, with the exception of Prn and BrkA, most of other known protective antigens such as PT, FHA, Fimbriae 2 and 3, and main pertussis virulence factors including TCT, LPS, CyaA and DNT always seem to escape detection by 1-D or 2-D immunoblotting [20], [28]. The explanation for this phenomenon might be due to the limited resolution capacity of 1-D and 2-D SDS-PAGE and mass spectrometric identification as well as to particular properties of these antigens, such as high molecular weight (FHA: 367 kDa and CyaA: 178 kDa), unusually basic proteins (Fimbriae 3: pI 9.10) and too low protein concentration (PT) [28].

### 4.1 Known *B.p* immunoreactive antigens

ACVs containing Prn can provide more effective protection against pertussis than ACVs without Prn [29]. Many studies from vaccine trials have provided abundant evidence that Prn serves as the most important adhesin playing an essential role in *B.p* adherence to host cells [30], [31]. In addition, Prn elicits stronger and more long-lasting antibody responses than other protective antigens such as PT and FHA, and thus it might be the protective antigen primarily recognized by immunoblot assays [32]. At the molecular level, Prn contains two repeat regions, GGxxP and PQP repeats, both of which have been identified as B-cell epitopes and elicit strong antibody responses in both humans and mice [33]. Like Prn, surface-exposed BrkA belongs to the same autotransporter family. The members of the autotransporter family include adhesins (FHA and Prn), toxins (virulence-activated genes 8, Vag8), invasins (TcfA) and proteases (Autotransporter subtilisin-like protease, SphB1). The essential function of these autotransporters is to direct their own export to the outer membrane or extracellular space. In addition to its main role in resistance of *B.p* to human immune serum complement-mediated killing, BrkA has been reported to contribute to the adherence and invasion of *B.p* to host cells in vitro and in vivo in a murine model of respiratory infection [34], [35]. Two porins, OmpP and OmpQ, are the predominant integral outer membrane proteins of *B. p* [36], [37]. Previous studies had shown that OmpP should be regarded as a potentially suitable subunit vaccine component, for it can not only serve as a highly conserved adhesin mediating adherence of *B.p* or *B.parapertussis* (*B.pp*) to human bronchial epithelial cells in vitro but also induce long-term high titers of bactericidal antibodies in immunized mice [38], [39]. Although the exact function of the Bvgi phase remains to be determined, this phase where conditions may be intermediate between the host environment and the environment outside appears to play a vital role in respiratory transmission [40]. BipA, the typical Bvgi-phase-specific gene, is highly similar to many well-characterized bacterial adhesins, intimins and invasins, and it might be associated with initial adherence and colonization of *B.p* to the respiratory tract of human hosts [41]. Thus, it was not surprising that BipA was found to be recognized by the human immune system and identified as a human immunoreactive protein of *B.p* in this work. In addition to Fimbriae 2 and 3, Prn, FHA and PT, which have multiple antigenic variations, BipA and OmpQ also have two different antigenic variations. In general, the variations in these antigens of currently circulating strains are distinct from those of vaccine strains of *B.p* [42], [43]. A plausible explanation is that these circulating strains are less affected by strong vaccine-driven selective pressures and are preferentially selected; thus, the antigenic divergence observed between circulating strains and vaccine strains may have gradually decreased the efficacy of these vaccines and led to the reemergence of pertussis. Consequently, BipA and OmpQ could be used as novel potential protective antigen candidates. Furthermore, GroEL has been previously used as a major target dominantly recognized by the immune system of infants vaccinated with WCV [44]. As one main component of the type IV secretion system of *B.p*, Not only was PtlF identified in the Surfaceome of *B. p*, but PtlE was also previously described to be immunodetected in B. p whole-cell lysates by using a specific mouse polyclonal antibody against recombinant PtlF protein [45].

### 4.2 Novel *B.p* immunoreactive proteins previously identified in other pathogens

Because of their wide immunogenicity among at least nine different pathogens, Elongation factor EF-Tu, EF-Ts and GAPDH represent the most typical examples of commonly conserved antigenic proteins. For instance, EF-Tu, a very abundant protein, has been shown to be highly immunogenic in *Anaplasma marginale* [46], *Borrelia hermsii* [47], *chlamydia trachomatis* [48], *Bacillus anthracis* [49], *Clostridium perfringens* [50], *Shigella flexneria* [51], *staphylococcus epidermidis* [52], *Francisella tularensis* [53], [54], [55] and *Bartonella quintans* [56]. In addition, it has been reported that cell surface-associated EF-Tu of many distinct bacteria show multiple activities binding to various mammalian proteins including mucin (*Lactobacillus johnsonii*) [57], fibrinogen (*Mycoplasma pneumoniae*) [58], plasminogen and factor H (*Pseudomonas aeruginosa*) [59]. Due to these biological binding activities, EF-Tu has been identified as an immunodominant surface antigen in *Anaplasma marginale*, *Staphylococcus aureus, Bacillus cereus, Mycobacterium chelonae* and *Actinobacillus pleuropneumoniae*, and it has strong potential to be a promising vaccine candidate due to its ability to induce high-level host immune responses [15], [60], [61], [62]. Interestingly, most of these newly identified *B.p* immunoreactive proteins are housekeeping enzymes that might be ideal diagnostic biomarkers or vaccine candidates due to their high antigenic conservation among different virulent strains of certain pathogens and even many distinct pathogens. As the best examples of this type of housekeeping enzyme, GAPDH has been shown to be highly immunogenic in more than nine pathogenic bacteria including *Paracoccidioides brasiliensis* [63], *Lactococcus garvieae* [64], *staphylococcus epidermidis* [52], *Streptococcus pneumoniate* [65], *Candida albicans* [66], [67], *Streptococcus suis* [16], *Francisella tularensis* [54], *Actinobacillus pleuropneumoniae* [15], *Haemonchus contortus* [68] and *Group A streptococcus* [69]. There are several experiments to show that GAPDH represents high levels of conserved immunogenicity, and hence, it has also been recognized as an important cross-strain protective antigen against *Schistosoma mansonii*, *Edwardsiella tarda*, *Onchocerca volvulus*, *Streptococcus pyogenes* and *Bacillus spp* [61], [65], [70], [71], [72]. The two typical examples of these uniquely antigenic proteins, PBP and Livj, have been already strictly identified as antigenic protein in *Shigella flexneri* and *Brucella abortus*, respectively [18], [73], [74]. PBP as surface-associated protein is mainly involved in the synthesis of the peptidoglycan layer of bacterial cell wall and is a main target of β-lactam antibiotics. Several investigations showed that PBPs have been required for virulence of many important human pathogens including *Mycobacterium tuberculosis*, *Group A Streptococcus*, *Group B streptococcus (GBS)* and *Streptococcus pneumonia* [75], [76], [77], [78]. LivJ representing the most abundant transport protein has been characterized in the extracellular proteomes of *E.coli* BL21 and W3110 strains, showing that LivJ could also serve as extracellular protein released outside the cell and thus recognized by host immune system [79]. In addition to presence in the Surfaceome and OMV proteome of *B. p*, putative ABC transport solute binding protein as a newly identified low-iron-induced protein displays differential protein expression levels between iron-excess and iron-starvation conditions, and it might be closely associated with *B.p* virulence [80]. Furthermore, putative ABC transport solute binding protein of *Neisseria meningitidis* has been regarded as a potentially new protective antigen for incorporation into multicomponent meningococcal vaccines; it was not only found to be exposed on the bacterial surface and well conserved across a range of meningococcal circulating strains, but also strongly reactive with sera from 31 convalescent young children infected with meningococcal disease [23], [81].

### 4.3 *B. p* immunoreactive proteins identified for the first time in this study

It is notable that members of the third group, putative 2-hydroexyacid dehydrogenase, Sbp, amino acid-binding periplasmic protein, putative exported solute binding protein and three hypothetical proteins (BP0250, BP2818 and BP3575) are reported to be immunogenic proteins for the first time in this study. Among them, BP2818 and putative exported solute binding protein were included in the OMV proteome of *B. p*, and only Sbp was identified as an immunogenic protein in both TMP and ECP fractions, indicating its highly reliable immunogenicity. The D-isomer specific 2-hydroxyacid dehydrogenase exhibits more than 50% sequence similarity to putative 2-hydroxyacid dehydrogenase which was identified as one of the antigenic proteins binding to serum antibody obtained from treponema pallidum-infected rabbits [82]. Pfam analysis revealed that BP2818 and BP3575 belong to the Lipoprotein_9 and ANF_receptor families, respectively. The bacterial Lipoprotein_9 family contains several antigenic members that may be involved in bacterial virulence, such as three highly immunoreactive antigens of *Pasteurella haemolytica*: plpA, -B and –C [83]. The ANF receptor family also includes extracellular ligand binding domains of a wide range of receptors. Similarly, Sbp, amino acid-binding periplasmic protein and putative exported solute binding protein are also identified as members of the SBP_bac_1, SBP_bac_3 and SBP_bac_7 families of bacterial extracellular solute-binding proteins, respectively, indicating their potential for secretion into the extracellular space.

Based on the theory of reverse vaccinology, the vast majority of these immunogenic proteins of the third group annotated as exposed on the cell surface by Gene Ontology and Pfam or included in the OMV proteome of *B. pertussis* should be the most likely suitable vaccine candidates because these proteins are easily recognized by the host immune system and therefore stimulate strong immune responses [21], [22], [23].

### 4.4 Human *versus* murine immunoproteome of *B.p* vaccine strains

Finally, apart from other similar findings such as those in *Helicobacter pylori* and *Francisella tularensis*, which showed high similarities in antigen recognition between humans and mice [54], [84], the comparison of the human immunoreactive proteins of *B. p* identified in our present work with previously identified murine immunoreactive proteins of *B. p* revealed many similarities but many more noticeable differences, reflecting heterogeneous immunoreactivity patterns between the human host and the murine model. Surprisingly, only the four human immunoreactive proteins, Prn, BrkA, GroEL and EF-Tu, were also immunodetected by serum from immunized or infected mice. Furthermore, the other human immunodominant antigens like OmpP, OmpQ, BipA and EF-Ts were not recognized by murine immune sera.

#### 4.4.1 Theoretical Expression Abundance and Gene Expression Profiling

**Figure 3:**
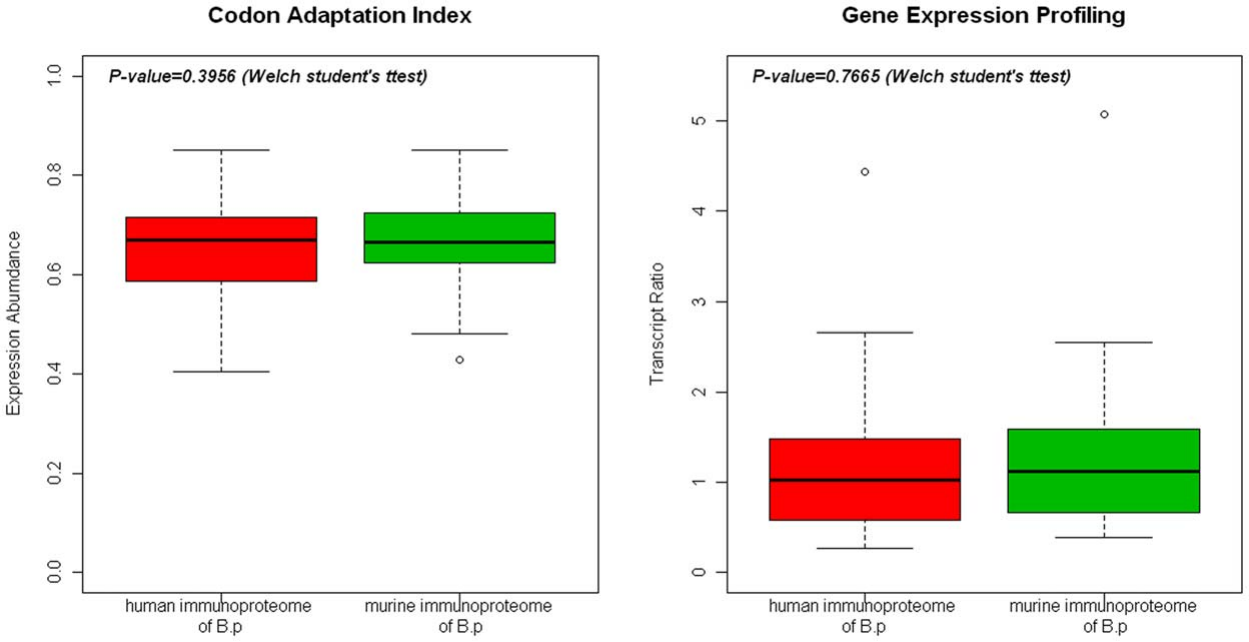
Comparison of the CAI values and gene expression profiling between the murine and human immunoproteomes of *B.pertussis* vaccine strains. Statistical analysis was performed with the R language.

In order to analyze the main reasons resulting in the heterogeneous immunoreactivity patterns, we further evaluated the differences in theoretical expression abundance and experimental gene expression profiling between the human and murine immunoreactive proteins of *B. p* using the JCAT tool and DNA microarray data (Shown in Fig. 3). The CAI values of these human and murine immunoreactive proteins of *B. p* ranged from 0.404 to 0.850 (mean: 0.641) and from 0.429 to 0.850 (mean: 0.664), respectively, and were not statistically different (*P*-value = 0.395). In accordance with the CAI values, transcript abundances of the human and murine immunoreactive proteins of *B. p* ranged from 0.27 to 4.44 (mean: 1.229) and from 0.385 to 5.00 (mean: 1.303), respectively, and were not statistically different (*P*-value = 0.7665). Thus, the results from both the CAI values and the gene expression profiling showed that the two groups of immunoreactive proteins shared same level of expression.

#### 4.4.2 Subcellular Localization

**Figure 4:**
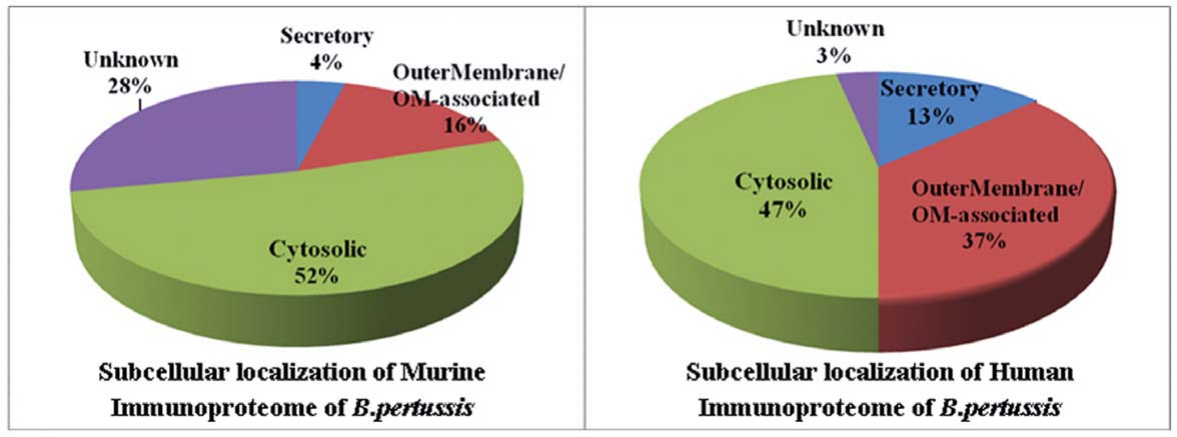
Subcellular localization distribution of these identified immunoreactive proteins in the murine and human immunoproteome of *B.pertussis* vaccine strains.

The sequences of these identified human immunoreactive proteins in *B.p* were analyzed using a combination of several algorithms and Gene Ontology in order to predict subcellular localization as described in our previous study with some modifications (See Table S1) [24]. A total of 37% (11/30) are predicted to localize to OuterMembrane/OM-associated, 47% (14/30) to cytoplasm, 13% (4/30) to secretory and 3% (1/30) are of unknown localizations. The subcellular localization of all murine immunoreactive proteins was as follows: 52% to cytoplasm, 16% to OuterMembrane/OM-associated, 4% to secretory and 28% to unknown localizations (See Fig. 4). Comparison of the subcellular localizations of the human and murine immunoreactive proteins demonstrated moderate similarities but somewhat disparate results mainly focused on the differential percentage of OuterMembrane/OM-associated proteins and proteins of unknown localization.

#### 4.4.3 Comparison of the human and murine immunoproteomes of *B.p* with the Surfaceome and OMV proteome of *B.p* vaccine strains

**Figure 5:**
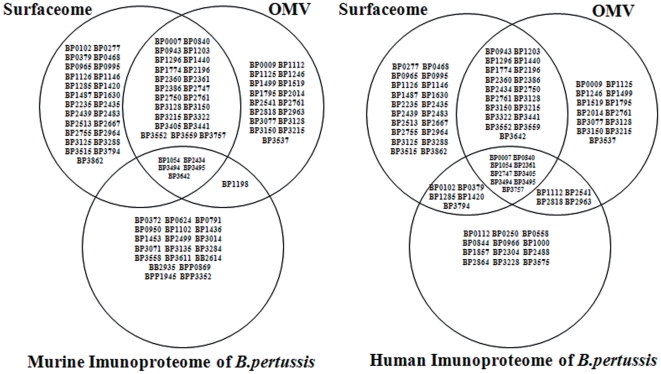
Venn diagram demonstrating the striking differences between the comparison of the murine or human immunoproteome with the Surfaceome or OMV proteome of *B.pertussis* vaccine strains, respectively. Detailed information is shown in Table S2.

In addition to comparing the two immunoproteomes to one another, we also compared the human and murine immunoproteomes of *B. p* with the previously reported Surfaceome and OMV proteome of *B. p* vaccine strains in Argentina, respectively (See Fig. 5 and Table S2) [25], [26]. A total of sixty percent (18/30) of the human immunoproteome of *B. p* were also present in the Surfaceome (46%; 14/30) and OMV proteome (43%; 13/30) of *B. p*, including all seven known pertussis antigens, several dehydrogenases, EF-Ts, EF-Tu, PBP and ABC transport solute-binding protein. By contrast, only 24% (6/25) of all murine immunoreactive proteins were present in the Surfaceome (20%; 5/25) and OMV proteome (24%; 6/25) of *B. p*, including Prn, BrkA, GroEL and serine protease.

As described above, the CAI values and gene expression profiling indicated that there was no correlation between the significant differences of the two immunoproteomes of *B. p* and the expression levels of these immunoreactive proteins. In addition, the differences of subcellular localization between the human and murine immunoreactive proteins were not remarkable. As these reasons above can be excluded, the precise reasons for the apparent differential immunoreactivity patterns might be due to the following determining factors:

1. Human immune system *versus* murine immune system. First, the most likely reason for this differential pattern is that certain *B. p* antigens may be recognized differently by the murine and human host immune systems, which contributes to the observed differences. To our knowledge, this is not for the first time that remarkably different immune responses between a mouse model and a human host have been reported. Keith Redhead had previously doubted the true value of the pertussis murine model for evaluating pertussis vaccine efficacy because the murine model and children vaccinated with WCV produced significantly differential antibody responses against several main protective antigens such as PT and FHA [11].

2. Pooled human and murine immune sera *versus* individual human and murine immune serum. With respect to vaccine and diagnostic applications, the biggest advantage of the pooled immune sera widely used in immunoproteomic analysis is to preferentially screen a small number of antigens with the highest level or the most conservative immunogenicity. However, other proteins eliciting weaker antibody responses or exclusively reactive with certain individual immune serum might be swamped. On the contrary, regarding inter-individual variation, increasing the number of individual immune serum leads to the identification of a larger number of antigens uniquely recognized by individual serum, and this might dramatically improve the similarities between the human and murine immunoproteomes of *B.p*. However, based on experimental goals, we and Emrah et al. chose pooled human and murine immune sera, respectively, instead of immune serum from a single individual. Therefore, using pooled human and murine immune sera might have contributed to the notable differences.

3. Chinese vaccine strain *versus* Turkey vaccine strain. Despite the high level of conservation of the whole genome among the majority of *B.p* strains [85], there is still a limited number of genomic divergences between *B. p* strains affecting certain key strain-specific protein and antigenic virulence factors [86], which may in part account for the discrepancy. For example, with the murine immunoproteome of *B. p* vaccine strain Saadet in Turkey, some newly identified antigens were more similar to those of two closed species of *B.p*, *B.pp* and *B. bronchiseptica* (*B.B*). In addition, Tohama I is the only *B. p* strain with a completely sequenced genome, and it is still used as the main *B. p* reference genome to date. However, it was recently found that several conserved genomic fragments among these *B. p* circulating isolates were deleted from Tohama I but present in *B.pp* and *B.B*, suggesting that Tohama I might not be a good representative strain of the *B. p* species [87]. The unsuitable reference genome of *B. p* might thus also account for the differences.

4. Subcellular fractions *versus* Whole cell fraction. Subcellular fractionation combining with high-resolution 2D-PAGE is usually an efficient approach significantly reducing the sample complexity from thousands of proteins in the whole cell fraction to hundreds of proteins in each subcellular fraction. In this study, in addition to two known protective antigens (Prn and BrkA) and two previously confirmed pertussis antigens (GroEL and EF-Tu) identified in the murine immunoproteome (whole cell fraction) and human immunoproteome (TMPs and ECPs), only another three confirmed antigens (OmpP, OmpQ and PtlF) were additionally identified in the human immunoproteome of *B.p*. However, none of other known protective antigens (PT, FHA, Fimbiae 2 and 3) and main virulence factors (Type-III secretion proteins, DNT, TCT, CyaA, TcfA, LPS and Vag8) was identified in the two immunoproteomes or Surfacome and OMV proteome of *B.p*. As described above, there is not big difference of the identified known pertussis antigens between the two immunoproteoms of *B.p*. On the contrary, total 11 (37%; 11/30) newly identified human immunoreactive proteins and only 3 (12%; 3/25) newly identified murine immunoreactive proteins were present in the Surfaceome or OMV proteome of *B.p*. The obvious differences between these newly identified human and murine immunoreactive proteins might be due to the reason that some low abundance proteins easily swamped by high abundance proteins in whole cell fraction can be effectively separated and immunodetected in TMPs and ECPs. Therefore, the sample preparation methodologies, which have significant effect on these newly identified human and murine immunoreactive proteins of *B.p* but not on these known pertussis antigens, should be also one of the main factors resulting in the apparent differences between the two immunoproteoms of *B.p*.

In summary, our results provide the first whole antigenic proteome profile of WCV and the repertoire of human antibody responses against WCV. These identified immunoreactive proteins notably include many previously unidentified antigens and they will pave the way for understanding the immunogenicity and pathogenesis mechanisms of *B. p.* Furthermore, they are regarded as suitable prognostic and diagnostic biomarkers as well as pathogenic targets for the development of new drugs and vaccines against pertussis. Importantly, this study also highlights the striking differences between the humoral immune responses of the mouse model and the human host.

## Supporting Information

Figure S1 2-D control immunoblot of ECPs and TMPs of *B. pertussis* Chinese WCV strain 58003.
(8.41 MB TIF)

Table S1 The human immunoreactive proteins identified in TMPs and ECPs of *B. pertussis* Chinese WCV strain 58003 by PMF.
(0.07 MB PDF)

Table S2 Comprehensive comparison of the human and murine immunoproteome of *B. pertussis* vaccine strains.
(0.10 MB PDF)
