# Supplementary figures and images for: Immunoproteomic Analysis of Human Serological Antibody Responses to Vaccination with Whole-Cell Pertussis Vaccine (WCV)
Source: PLoS One. 2010 Nov 9;5(11):e13915. doi: 10.1371/journal.pone.0013915 (PMC2976700; doi:10.1371/journal.pone.0013915)

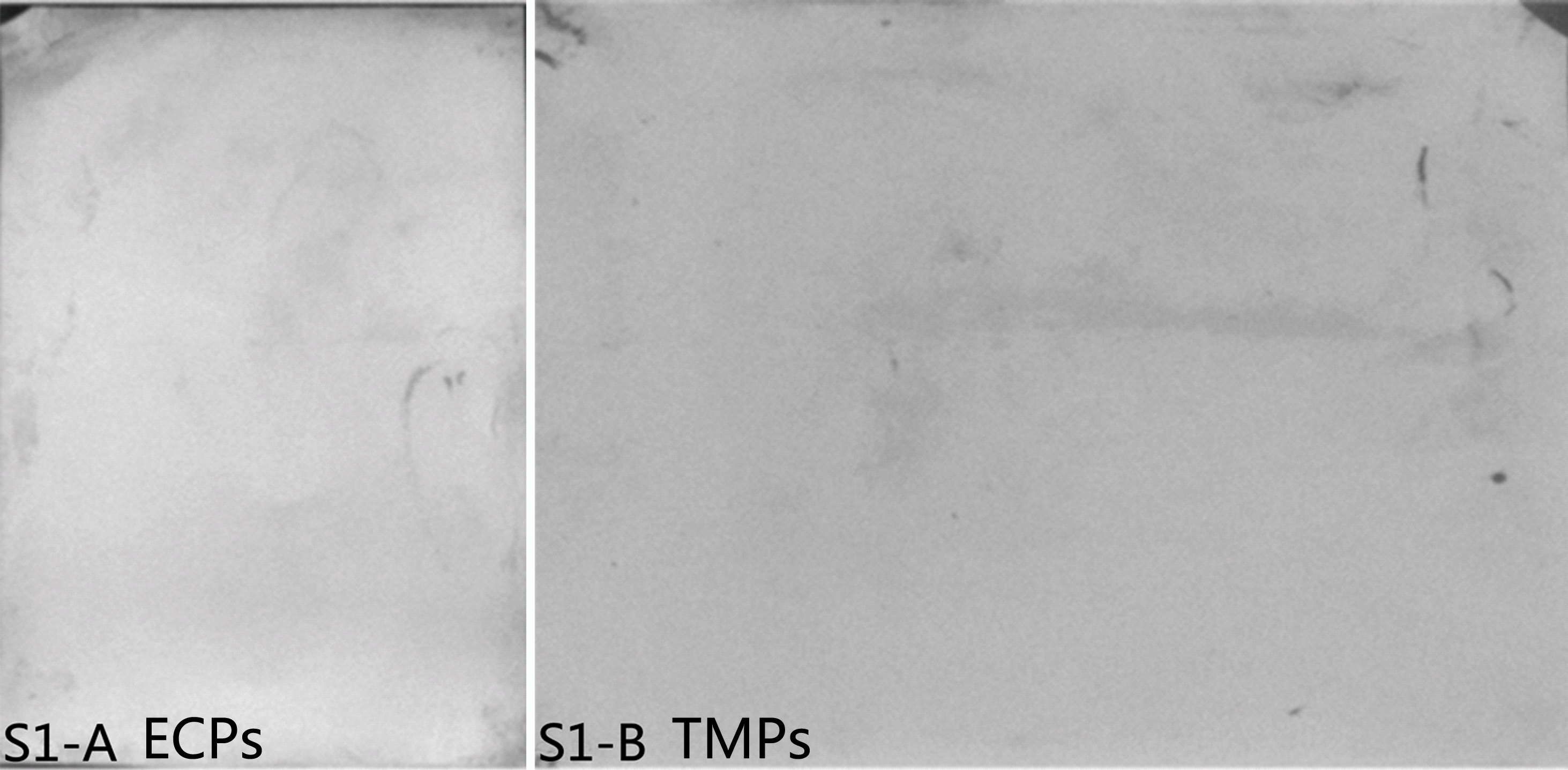

Supplement: Figure S1 — 2-D control immunoblot of ECPs and TMPs of B. pertussis Chinese WCV strain 58003. (8.41 MB TIF) [file pone.0013915.s001.tif]
