# Supplementary material for: Immunoproteomic Analysis of Human Serological Antibody Responses to Vaccination with Whole-Cell Pertussis Vaccine (WCV)
Source: PLoS One. 2010 Nov 9;5(11):e13915. doi: 10.1371/journal.pone.0013915 (PMC2976700; doi:10.1371/journal.pone.0013915)
Supplement: Table S2 — Comprehensive comparison of the human and murine immunoproteome of B. pertussis vaccine strains. (0.10 MB PDF) [file pone.0013915.s003.pdf]

Table S1: The human immunoreactive proteins identified in TMPs and ECPs of *B.pertussis* Chinese WCV strain 58003 by PMF

Spot ID	GI	Locus	Location	Protein Product	COG	Theoretical MW/pI	Experimental MW/pI	Mascot Score	Matched peptides	Sequence coverage
MP1	33592122	BP0966	OM-associated	sulfate-binding protein precursor (Sbp)	P	37913/7.83	37891/5.01	169	12	38%
MP2,12,16	33594369	BP3494	Outermembrane	serum resistance protein (BrkA)	MU	103377/6.62	103315/6.15	172	16	22%
MP3	33594289	BP3405	Outermembrane	outer membrane porin protein OmpQ (OmpQ)	M	39141/5.59	39117/5.17	198	13	41%
MP4	33592006	BP0840	Outermembrane	outer membrane porin protein precursor (OmpP)	M	41045/5.51	41020/4.07	209	14	51%
MP5	33591607	BP0379	Cytoplasm	putative L-lactate dehydrogenase (Ldh)	C	37214/5.59	37272/6.21	116	10	30%
MP6	33592912	BP1857	Cytoplasm	glutamate dehydrogenase (GdhA)	E	46323/5.97	46295/6.55	132	13	33%
MP7	33593353	BP2361	Cytoplasm	succinate dehydrogenase (SdhA)	C	64855/6.07	64815/6.39	101	11	20%
MP8	33592010	BP0844	Cytoplasm	NADH dehydrogenase delta subunit (NuoD)	C	47458/5.75	47398/6.00	157	15	34%
MP9	33592195	BP1054	Extracellular	pertactin precursor (Prn)	MU	93452/9.23	93396/6.69	102	12	26%
MP10,21	33594370	BP3495	Cytoplasm	chaperonin GroEL (GroEL)	O	57481/5.13	57446/4.55	192	16	26%
MP11	33593707	BP2747	OM-associated	putative ABC transport solute-binding protein	E	40652/6.17	40699/6.14	128	11	24%
MP13	33593471	BP2488	Cytoplasm	isocitrate dehydrogenase (Icd)	C	45793/5.71	45765/5.66	135	10	28%
MP15	33593298	BP2304	Cytoplasm	putative 2-hydroxyacid dehydrogenase	HE	33902/5.73	33882/5.09	179	12	56%
MP17	33594134	BP3228	Cytoplasm	putative septum site-determining protein (MinD)	D	29681/5.20	29663/4.99	88	7	23%
MP18	33594616	BP3757	Periplasm	putative ABC transporter, ATP-binding protein	Q	29624/5.26	29606/4.89	115	10	32%
MP20	33593524	BP2541	Cytoplasm	succinyl-CoA synthetase subunit beta (SucC)	C	40939/5.23	40913/4.65	151	13	25%
MP24	33593808	BP2864	Cytoplasm	putative alcohol dehydrogenase	CR	36683/5.94	36660/6.13	83	7	27%
MP25	33592150	BP1000	Cytoplasm	glyceraldehyde-3-phosphate dehydrogenase (GAPDH)	G	36284/6.37	36262/7.00	66	6	24%
MP26	33591361	BP0102	Periplasm	putative penicillin-binding protein precursor (PBP)	M	44879/7.80	44851/7.08	218	14	29%
MP27	33593772	BP2818	OM-associated	hypothetical protein BP2818	P	28742/7.82	28725/6.20	147	11	48%
MP28	33594649	BP3794	Unknown	putative bacterial secretion system protein (PtlF)	U	29471/6.30	29453/6.44	90	7	35%

MP29	33591491	BP0250	OM-associated	hypothetical protein BP0250	S	34534/7.66	34513/7.04	90	7	35%
MP30	33591370	BP0112	Cytoplasm	cystathionine beta-lyase (MetC)	E	43323/5.90	43296/5.31	107	9	31%
SP1	33594369	BP3494	Outermembrane	serum resistance protein (BrkA)	MU	103377/6.62	103315/5.89	255	23	25%
SP2	33592195	BP1054	Extracellular	pertactin precursor (Prn)	MU	93452/9.23	93396/6.50	217	19	35%
SP3	33594444	BP3575	Periplasm	hypothetical protein BP3575	E	43185/5.92	43159/5.60	109	12	29%
SP4	33591281	BP0007	Cytoplasm	elongation factor Tu (EF-Tu)	-	42916/5.34	42889/5.23	138	12	34%
SP5	33592409	BP1285	OM-associated	leu/ile/val-binding protein precursor (LivJ)	-	39608/6.33	39583/6.37	96	9	35%
SP6	33592006	BP0840	Outermembrane	outer membrane porin protein precursor (OmpP)	M	41045/5.51	41020/5.14	155	12	35%
SP7	33592122	BP0966	OM-associated	sulfate-binding protein precursor (Sbp)	P	37913/7.83	37891/6.55	203	15	60%
SP8	33592249	BP1112	Outermembrane	putative outer membrane ligand binding protein (BipA)	-	137194/6.26	137111/5.91	309	28	37%
SP9	33591762	BP0558	OM-associated	amino acid-binding periplasmic protein	ET	36140/6.64	36118/6.27	157	13	36%
SP10	33592518	BP1420	Cytoplasm	elongation factor Ts (EF-Ts)	J	30904/5.31	30885/5.11	255	18	60%
SP11	33593898	BP2963	OM-associated	putative exported solute binding protein	Q	40317/8.26	40291/6.76	162	14	32%
